# Targeting fibroblast activation protein in newly diagnosed squamous cell carcinoma of the oral cavity – initial experience and comparison to [^18^F]FDG PET/CT and MRI

**DOI:** 10.1007/s00259-021-05422-z

**Published:** 2021-05-29

**Authors:** Christian Linz, Roman C. Brands, Olivia Kertels, Alexander Dierks, Joachim Brumberg, Elena Gerhard-Hartmann, Stefan Hartmann, Andreas Schirbel, Sebastian Serfling, Yingjun Zhi, Andreas K. Buck, Alexander Kübler, Julian Hohm, Constantin Lapa, Malte Kircher

**Affiliations:** 1 Department of Oral and Maxillofacial Plastic Surgery, University Hospital of Würzburg, Pleicherwall 2, 97070 Würzburg, Germany; 2 Comprehensive Cancer Center Mainfranken, University Hospital of Würzburg, Josef-Schneider-Str. 6, 97080 Würzburg, Germany; 3 Institute for Diagnostic and Interventional Radiology, University Hospital of Würzburg, Oberdürrbacherstr. 6, 97080 Würzburg, Germany; 4 Department of Nuclear Medicine, University Hospital of Würzburg, Oberdürrbacherstr. 6, 97080 Würzburg, Germany; 5 Nuclear Medicine, Medical Faculty, University of Augsburg, Stenglinstraße 2, 86156 Augsburg, Germany; 6 Department of Nuclear Medicine, University Hospital of Freiburg, Hugstetter Straße 55, 79106 Freiburg, Germany; 7 Department of Pathology, University of Würzburg, Josef-Schneider-Str.2, 97080 Würzburg, Germany; 8 Department of Otorhinolaryngology, University Hospital of Würzburg, Josef-Schneider-Str. 11, 97080 Würzburg, Germany

**Keywords:** Molecular imaging, Fibroblast activation protein, Head and neck cancer, PET

## Abstract

**Purpose:**

While [^18^F]-fluorodeoxyglucose ([^18^F]FDG) is the standard for positron emission tomography/computed tomography (PET/CT) imaging of oral squamous cell carcinoma (OSCC), diagnostic specificity is hampered by uptake in inflammatory cells such as neutrophils or macrophages. Recently, molecular imaging probes targeting fibroblast activation protein α (FAP), which is overexpressed in a variety of cancer-associated fibroblasts, have become available and might constitute a feasible alternative to FDG PET/CT.

**Methods:**

Ten consecutive, treatment-naïve patients (8 males, 2 females; mean age, 62 ± 9 years) with biopsy-proven OSCC underwent both whole-body [^18^F]FDG and [^68^Ga]FAPI-04 (FAP-directed) PET/CT for primary staging prior to tumor resection and cervical lymph node dissection. Detection of the primary tumor, as well as the presence and number of lymph node and distant metastases was analysed. Intensity of tracer accumulation was assessed by means of maximum (SUV_max_) and peak (SUV_peak_) standardized uptake values. Histological work-up including immunohistochemical staining for FAP served as standard of reference.

**Results:**

[^18^F]FDG and FAP-directed PET/CT detected all primary tumors with a SUV_max_ of 25.5 ± 13.2 (FDG) and 20.5 ± 6.4 (FAP-directed) and a SUV_peak_ of 16.1 ± 10.3 ([^18^F]FDG) and 13.8 ± 3.9 (FAP-directed), respectively. Regarding cervical lymph node metastases, FAP-directed PET/CT demonstrated comparable sensitivity (81.3% vs. 87.5%; *P* = 0.32) and specificity (93.3% vs. 81.3%; *P* = 0.16) to [^18^F]FDG PET/CT. FAP expression on the cell surface of cancer-associated fibroblasts in both primary lesions as well as lymph nodes metastases was confirmed in all samples.

**Conclusion:**

FAP-directed PET/CT in OSCC seems feasible. Future research to investigate its potential to improve patient staging is highly warranted.

**Supplementary Information:**

The online version contains supplementary material available at 10.1007/s00259-021-05422-z.

## Introduction

Oral squamous cell carcinoma (OSCC) is the sixth most common tumor entity worldwide and the ninth most frequent cause of death [1, 2]. With an estimated incidence of about 275,000 cases per year, it accounts for more than 90% of all malignancies of the oral cavity [3, 4]. Since the presence of cervical lymph node (LN) metastasis is one of the most relevant negative prognostic factors [5–8] and detection of distant metastases shifts therapy from a curative to a palliative approach [9], accurate tumor staging is of paramount importance for adequate treatment choice and estimation of prognosis [10–12].

The utility of positron emission tomography/computed tomography (PET/CT) with [^18^F]-fluorodeoxyglucose ([^18^F]FDG) for staging malignancies of the head and neck is well documented [13–18]. However, specificity of [^18^F]FDG is hampered by uptake in inflammatory cells such as neutrophils or macrophages [19]. Since inflammatory processes are very common in head and neck tumors [20], there is an urgent need for a more specific alternative to [^18^F]FDG to further improve preoperative staging and therapy of OSCC.

Cancer-associated fibroblasts (CAF) are located within the tumor stroma and modulate the tumor microenvironment by secretion of cytokines, chemokines and growth factors. In OSCC, tumor invasion is promoted by the communication between CAF and tumor cells [21, 22]. A particularity of CAF is the overexpression of fibroblast activation protein α (FAP) which corresponds to a type II transmembrane glycoprotein and acts as a serine protease of the dipeptidyl-peptidase family [22, 23]. FAP overexpression on CAF of the tumor microenvironment has been confirmed in more than 90% of epithelial carcinomas, including malignancies of the breast, lung, colon and head and neck [22, 24, 25]. In contrast, FAP is nearly absent in normal adult tissues [26, 27], rendering it a rather specific target for tumor imaging. Recently, FAP-targeted radioligands that act as FAP inhibitors (FAPI) have become available for PET/CT imaging [28]. And whereas its initially anticipated excellent specificity has been partially relativized by high tracer uptake in non-malignant inflammatory processes [29–33], FAP-directed PET/CT might prove a suitable tool for staging of OSCC.

The aim of this pilot study was to investigate the feasibility of staging newly diagnosed, treatment-naïve OSCC patients using [^68^Ga]FAPI-04 (FAP-directed) PET/CT, and to compare its diagnostic performance with that of [^18^F]FDG PET/CT and cervical magnetic resonance imaging (MRI).

## Methods

### Patients and staging

Between July 2018 and March 2020, ten consecutive patients (8 males, 2 females; mean age, 62 ± 9 years) with newly diagnosed, biopsy-proven, treatment-naïve OSCC underwent whole-body [^18^F]FDG PET/CT and cervical MRI for primary staging. In addition, FAP-directed PET/CT was performed within a median interval of 4 days (range, 2 – 16 days). Within two weeks after imaging all patients underwent surgery according to institutional standards of care. Surgical treatment consisted of resecting the local primary tumor and performing a selective (levels I–III, levels I–III and Va, or levels II, III, and Va) or a complete neck dissection of levels I–V according to Robbins’ classification [34]. Resected primary tumors and lymph nodes were histopathologically assessed for the presence of tumor cells.

^68^ Ga-FAPI-04 was administered on a compassionate use basis, in compliance with §37 of Declaration of Helsinki and the German Medicinal Products Act, AMG §13 2b. Written, informed consent was obtained from all subjects. Data analysis was revealed to the local institutional review board of the University of Würzburg that approved of this study (2,020,100,201).

### PET/CT and MR imaging

Imaging was performed on an integrated PET/CT scanner (Siemens Biograph mCT 64, Siemens Healthineers, Knoxville, USA). Prior to [^18^F]FDG PET/CT imaging, patients fasted for at least 4 h with blood glucose levels below 160 mg/dl; for FAP-directed imaging no dedicated preparation was required. Mean injected activity was 269 ± 43 MBq (range, 204 – 317 MBq) for [^18^F]FDG, and 119 ± 34 MBq (range, 66 – 168 MBq) for [^68^ Ga]FAPI-04, respectively. There were no adverse or clinically detectable pharmacological effects in any of the 10 subjects. No significant changes in vital signs or the results of laboratory studies or electrocardiograms were observed. Whole-body (top of the skull to knees) PET scans were performed one hour after administration of the radiopharmaceutical.

PET emission data were acquired in 3D-mode with a 200 × 200 matrix with 2 min emission time per bed position from the vertex of the skull to the proximal thighs. As part of [^18^F]FDG PET/CT, transmission data were acquired using contrast-enhanced spiral CT (dose modulation with a quality reference of 180 mAs, 120 kV, a 512 × 512 matrix, 5 mm slice thickness, increment of 30 mm/s, rotation time of 0.5 s, and pitch index of 1.4). Furthermore, a dedicated acquisition of the head and neck with one bed position, 3 min emission time, and contrast-enhanced CT was performed (180 mAs, 120 kV, a 512 × 512 matrix, 3 mm slice thickness, increment of 30 mm/s, rotation time of 1.0 s, and pitch index of 0.9). For anatomical co-registration of the FAP-directed scan, a non-contrast enhanced CT protocol with Care Dose 4D and a quality reference of 80 – 120 mAs was used.

All PET data were reconstructed iteratively (3 iterations, 24 subsets, Gaussian filtering of 2.0 mm full width at half maximum) with attenuation correction using dedicated standard software (HD. PET, Siemens Esoft, Siemens Healthineers, Erlangen, Germany).

MRI scans of the head and neck area were acquired using a 1.5 T scanner (Siemens Magnetom Avanto fit, Siemens Healthcare, Erlangen, Germany) or a 3.0 T scanner (Siemens Magnetom Prisma or Skyra, Siemens Healthcare, Erlangen, Germany) with a 64-channel head/neck coil for signal reception. Various T1, T2 and diffusion weighted sequences were done with and without contrast agent (Dotagraf; Jenapharm GmbH & Co. KG, Jena, Germany).

### Image analysis

Three experienced nuclear medicine physicians (AD, OK and CL) blinded to all clinical data independently rated all PET/CT scans in random order and at two time points on a syngo.via workstation (Siemens Healthineers, Erlangen, Germany). Foci of increased tracer uptake with reference to normal tissue and blood pool and/or the presence of morphological alterations on CT images were recorded as being positive for tumor involvement. The localization, expansion, and infiltration of osseous structures as well as the presence and number of nodal metastases were recorded for each cervical lymph node level. Lymph node levels were assessed according to the imaging-based nodal classification [12]. Furthermore, whole-body scans were evaluated for distant nodal and organ metastasis and for secondary malignancies. Any initial difference of rating between the three readers was resolved by subsequent consensus reading. Maximum and peak (defined as average activity concentration within a 1 cm^3^ spherical VOI centered on the “hottest” voxel) standardized uptake values (SUV_max_ and SUV_peak_) of the primary tumor, and suspected LN metastases were measured.

MRI scans were evaluated by two experienced board-certified radiologists (AD and OK) according to previously published methods [35, 36]. Lymph node levels were assessed according to the imaging-based nodal classification [37, 38].

### Histopathological analysis

Specimens were analyzed with regard to tumor size, lymph node metastases, lymph vessel- and venous invasion, perineural infiltration, resection status, and tumor grading according to the TNM classification of OSCC [12].

Immunohistochemistry (IHC) for FAP (antibody ab207178, Abcam, 1:250) was performed on formalin-fixed paraffin-embedded tissue slides according to standard IHC protocols. Immunostaining was scored as previously described [39, 40]. In brief, semi-quantitative analysis of stromal staining was scored as 0 (no staining), 1 + (1—10%), 2 + (11—50%), and 3 + (51—100% stromal staining). Tumor-free lymph nodes served as negative controls.

### Statistical analysis

Statistical analyses were performed using the statistics software SPSS (version 24.0; IBM Armonk, US) and R (R v3.6.1, http://www.R-project.org/). Quantitative values are displayed as mean ± standard deviation or median and range, as appropriate. Spearman’s Rho correlation analysis was used to compare uptake of both tracers in corresponding tumors/lymph nodes. McNemar’s exact test was used to compare sensitivity and specificity of both tracers and MRI. *P* values ≤ 0.05 were considered statistically significant.

## Results

### Tumor localization and staging

Five patients suffered from a primary tumor of the floor of the mouth (two central, three lateral), two subjects from a unilateral primary of the tongue. The mandibular mucosa was affected in two instances, the maxillary in the remaining case. Mean size of the primary lesion was 28.3 ± 12.9 mm (range, 11.0 – 46.0 mm), resulting in a T-stage of T2 in five patients, T3 in two subjects and T4 in the remaining three patients, respectively. A total of 434 lymph nodes were resected, of which 3.7% (16/434) harbored metastatic disease in seven patients. The mean size of LN metastases was 11.4 ± 9.7 mm (median, 9 mm; range, 2.0 – 43.0 mm). Neither distant hematologic spread nor second malignancy was observed in this cohort. Details are given in Table 1.

**Table 1:** Patients’ characteristics

Pat #	Age	Sex	Primary Tumor					Lymph nodes			Distant Mets	
			Location	Side	Size [mm]	Osseous arrosion /infiltration	T-Stage	Resected	Mets	N-Stage		M-Stage
1	64	M	alveolar process of the mandible	bilateral	15	yes	2	59	4	3b	none	0
2	54	M	floor of the mouth	right	42	yes	3	50	3	3b	none	0
3	66	M	floor of the mouth	bilateral	32	no	2	36	0	0	none	0
4	49	M	floor of the mouth	right	34	no	2	53	1	1	none	0
5	56	F	tongue	right	46	no	3	47	1	2a	none	0
6	60	M	floor of the mouth	right	21	no	2	23	0	0	none	0
7	58	M	floor of the mouth	bilateral	27	yes	4	49	1	2a	none	0
8	80	F	maxillary mucosa	right	42	yes	4	46	4	2b	none	0
9	62	M	tongue	right	13	no	2	14	0	0	none	0
10	72	M	alveolar process of the mandible	right	11	yes	4	57	2	3b	none	0

F, female; LN, lymph node; Mets, metastases; M, male; Pat, patient

### [^18^F]FDG PET/CT

On a per-patient analysis [^18^F]FDG PET/CT detected all primary tumors with a mean SUV_max_ and SUV_peak_ of 25.5 ± 13.2 (range, 9.6 – 48.1) and 16.1 ± 10.3 (range, 5.1 – 34.0), respectively. 85.7% (6/7) of patients with LN metastases were correctly identified.

On a per-lesion basis [^18^F]FDG PET/CT revealed 87.5% (14/16) of LN metastases with a mean SUV_max_ and SUV_peak_ of 14.9 ± 12.3 (range, 2.9 – 40.6) and 8.3 ± 6.8 (range, 1.8 – 25.2), respectively, while the remaining 2/16 (12.5%; patients #4 and #8) were false negative. Of note, the LN metastases missed by [^18^F]FDG PET/CT were each only five mm or less in size. Three out of 17 LNs that were rated as PET-positive turned out to be false positive. Thus, sensitivity and specificity for detection of LN metastases were 87.5% and 81.3%, respectively. Details are given in Table 2.

**Table 2:** Individual PET results and immunohistochemical FAP expression of lymph node metastases

Pat#	LN [Mets/resected]	Location [Side / Level]	Size [mm]	[^18^F]FDG PET		FAP-directed PET		IHC
				SUV_max_	SUV_peak_	SUV_max_	SUV_peak_	FAP CAF
1	4 / 59	R / Ib	11	10	3	8	5	2 +
		R / Ia	5	3	2	3	2	2 +
		L / Ia	2	3	2	n/d	n/d	3 +
		L / Ib	6	7	4	5	3	3 +
2	3 / 50	R / Ib	11	8	5	8	5	2 +
		R / Ib	9	5	3	6	4	2 +
		R / IIa	12	7	5	8	6	3 +
3	0 / 36							
4	1 / 53	R / IIa	5	n/d	n/d	n/d	n/d	2 +
5	1 / 47	R / IIa	43	21	13	13	8	2 +
6	0 / 23							
7	1 / 49	R / Ib	9	5	5	6	4	3 +
8	4 / 46	R / Ib	22	34	17	20	11	3 +
		R / Ib	5	n/d	n/d	n/d	n/d	2 +
		R / IIa	8	16	8	8	6	1 +
		R / IIb	8	20	12	8	6	2 +
9	0 / 14							
10	2 / 57	R / Ib	12	41	25	20	14	3 +
		R / IIa	15	29	12	25	9	2 +

CAF, cancer-associated fibroblasts; FAP, fibroblast activation protein; IHC, immunohistochemistry; L, left; n/d, not detected; R, right; LN, lymph node; Mets, metastases; SUV, standardized uptake value

### FAP-directed PET/CT

On a per-patient basis FAP-directed PET/CT correctly identified all primary tumors with a mean SUV_max_ and SUV_peak_ of 20.8 ± 6.4 (range, 7.0 – 29.1) and 13.8 ± 3.9 (range, 5.5 – 18.6), respectively, which was comparable to the values derived for FDG PET (r_s_ = 0.56, *P* = 0.09 and r_s_ = 0.84, *P* = 0.002, respectively). Six out of seven patients with LN metastases were correctly identified.

**Fig. 1:**
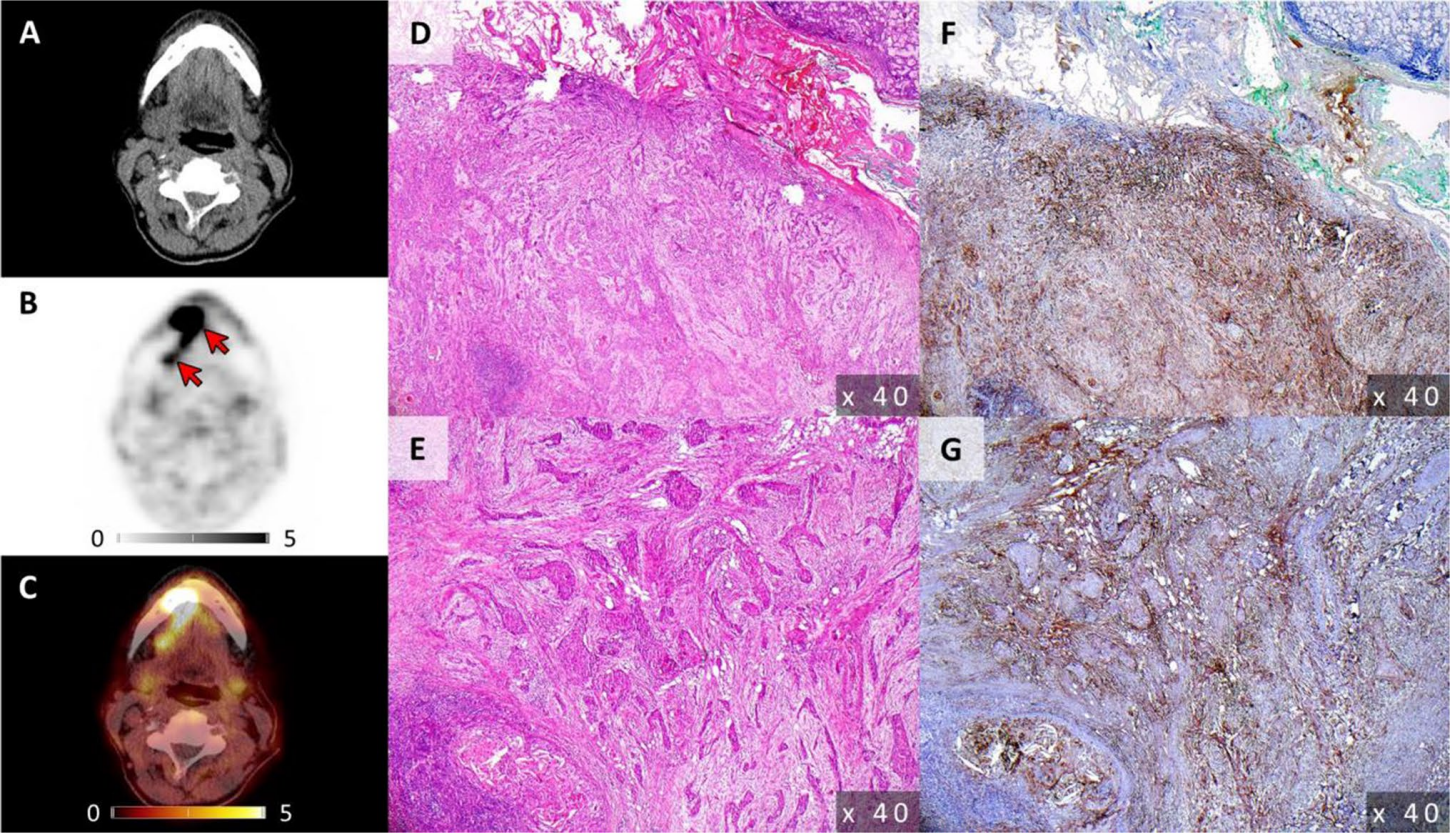
Example of FAP-directed imaging and respective immunohistochemistry of both primary tumor and cervical lymph node metastasis in a patient with newly diagnosed, treatment-naïve oral squamous cell carcinoma. Computed tomography (CT, **A**), fibroblast activation protein (FAP)-directed positron emission tomography (PET, **B**) as well as hybrid PET/CT imaging (**C**) in a patient with newly diagnosed, treatment-naïve squamous cell carcinoma of the alveolar process of the mandible (patient #1) depicts both the primary tumor as well as an adjacent cervical lymph node metastasis (arrows). Histological work-up (**D**-**G**) including immunohistochemistry for FAP (**F**, **G**) could confirm presence of FAP-positive disease in both instances (primary tumor: **D**, **F**; lymph node metastasis: **E**, **G**)

**Fig. 2:**
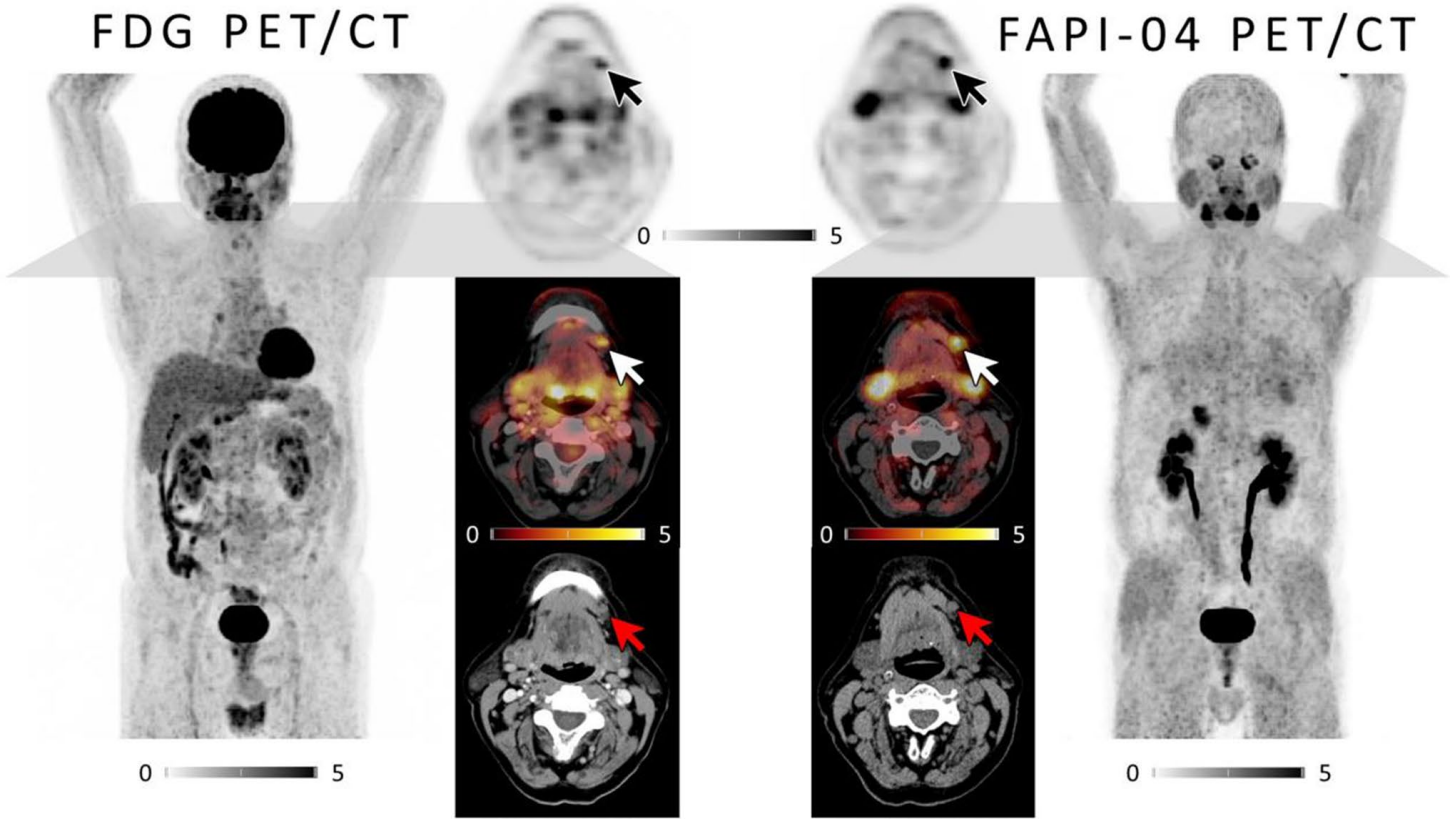
Comparison of [^18^F]FDG and FAP-directed [^68^ Ga]FAPI-04 PET/CT in a patient with newly diagnosed, treatment-naïve oral squamous cell carcinoma and a cervical lymph node metastasis. Maximum Intensity Projections (MIP, outer columns) as well as axial PET (top), fused PET/CT (middle) and CT (bottom) slices of [^18^F]FDG (left) and [^68^ Ga]FAPI 04 (right) PET scans in a patient with newly diagnosed, treatment-naïve squamous cell carcinoma of the alveolar process of the mandible (patient #1). Whereas both tracers detect the regional lymph node metastasis in cervical level Ib, FAP-directed imaging offers higher tracer uptake. Of note, neither tonsils nor other lymph nodes are [^68^ Ga]FAPI-04 PET-positive in this example

On a per-lesion basis FAP-directed PET/CT detected 81.3% (13/16) of LN metastases with a mean SUV_max_ and a SUV_peak_ of 10.7 ± 6.9 (range, 3.1 – 25.2) and 6.4 ± 3.3 (range, 2.4 – 13.8), respectively. The remaining 3/16 LN metastases (18.8%; patients #1, #4 and #8) with a respective LN size of 2 mm (patient #1) and 5 mm (patients #4 and #8) were missed. One of 14 LNs that were rated to be suspicious for metastatic disease turned out to be false positive, resulting in a sensitivity and specificity for the detection of LN metastases of 81.3% and 93.3%, respectively. Examples of FAP-directed imaging results are displayed in Figs. 1 and 2.

### MRI

On a per-patient basis MRI correctly identified all primary tumors and six out of seven patients with LN metastases. Of note, one of the patients with lymph node metastasis (patient #4) was rated positive due to a false positive LN, while the true metastasis was rated FN.

On a per-lesion basis MRI detected 50.0% (8/16) of LN metastases. The remaining 8/16 LN metastases (50.0%; patients #1, #4, #7, #8 and #9) with a respective LN size between 2 and 12 mm were missed. Five of 13 LNs that were rated to be suspicious for metastatic disease turned out to be false positive, resulting in a sensitivity and specificity for the detection of LN metastases of 50.0% and 61.5%, respectively. However, it must be noted that there were significant imaging artefacts in four subjects (#4, #5, #7 and #10), particularly due to metal implants (patients #4, #5 and #7) and patient movement (patients #4 and #10).

### Immunohistochemistry

All primary tumors and all lymph node metastases showed a variable target expression with 15/16 cases demonstrating positive FAP immunostaining in > 10%-50% (score 2 + ; 56%) and > 50% of surrounding stromal cells (score 3 + ; 38%). FAP expression was also confirmed in the metastases missed by FAP-directed PET/CT (patients #1, #4 and #8). Individual IHC results for FAP for both primary lesions and LN metastases are given in Table 3.

**Table 3:** Results of N-Staging according to [^18^F]FDG PET/CT, [^68^ Ga]FAPI-04 PET/CT and MR imaging

	Histo	[^18^F]FDG PET/CT					[^68^ Ga]FAPI-04 PET/CT					MRI				
Pat #	LN (Mets/total)	TP	FP	TN	FN	Effect on N-Staging	TP	FP	TN	FN	Effect on N-Staging	TP	FP	TN	FN	Effect on N-Staging
1	4 / 59	4	0	55	0	→	3	0	55	1	→	1	0	55	3	↓
2	3 / 50	3	1	46	0	→	3	0	47	0	→	3	2	45	0	→
3	0 / 36	-	0	36	-	→	-	0	36	-	→	-	0	36	-	→
4	1 / 53	0	1	51	1	→ *	0	0	52	1	↓	0	1	51	1	→ *
5	1 / 47	1	0	44	0	→	1	1	45	0	→	1	1	45	0	↑
6	0 / 23	-	1	22	-	↑	-	0	23	-	→	-	0	23	-	→
7	1 / 49	1	0	48	0	→	1	0	48	-	→	0	0	48	1	↓
8	4 / 46	3	0	42	1	→	3	0	42	1	→	2	0	42	2	↓
9	0 / 14	-	0	14	-	→	-	0	14	-	→	-	0	14	-	→
10	2 / 57	2	0	55	0	→	2	0	55	0	→	1	1	54	1	→ *
	**16/434**	**14/16**	**3/418**	**413/418**	**2/16**	**1/10**	**13/16**	**1/418**	**417/418**	**3/16**	**1/10**	**8/16**	**5/418**	**413/418**	**8/16**	**5/10**

^*^ unchanged, but due to false positive lymph node; ↑, overstated; → , unchanged; ↓, understated; FAPI, fibroblast activation protein inhibitor; FDG, fluorodesoxyglucose; FN, false negative; FP, false positive; Histo, histopathology; LN, lymph node(s); Mets, metastases; MRI, magnetic resonance imaging; Pat #, patient number; TN, true negative; Total, total number of resected lymph nodes; TP, true positive

In addition to the reactivity in CAF, we also observed a reactivity of variable quantity and intensity, but mostly weaker than in the CAF, in the cytoplasm and / or on the cell surface of the tumor cells in a subset of samples.

## Discussion

In this pilot study in patients with newly diagnosed, treatment-naïve OSCC, CAF-directed molecular imaging showed promise as a feasible diagnostic alternative to standard [^18^F]FDG PET/CT.

Due to the high glucose consumption of tumor cells, [^18^F]FDG is the reference tool for molecular imaging of squamous cell carcinoma of the head and neck and has proven its clinical value in a number of trials with high sensitivity and acceptable specificity [13–18, 41]. A recent multi-center trial including 23 different imaging sites in the US prospectively evaluated the value of [^18^F]FDG PET/CT in patients with newly diagnosed, first-time, head and neck squamous cell carcinoma and confirmed the high negative predictive value of this imaging modality [42].

In our cohort, [^18^F]FDG PET/CT detected all primary tumors and most lymph node metastases with a sensitivity of 87.5% and a specificity of 81.3%, which is in line with the body of literature including a recent meta-analysis [16, 42, 43]. It is worth mentioning that the lymph node metastases missed by [^18^F]FDG PET/CT had a size of 5 mm or smaller, which is at the limit of the system’s technical image resolution.

Molecular imaging with [^68^ Ga]-FAPI-04 PET/CT demonstrated identical primary tumor detection. Regarding metastases a slightly lower sensitivity (81.3%) as well as a marginally higher specificity (93.3%) for cervical lymph node involvement was observed in comparison to [^18^F]FDG PET/CT. PET signal intensity as assessed by SUV_max_ varied between 7.0 and 29.1 and is in line with recent studies that investigated the diagnostic performance of [^68^ Ga]FAPI-04 PET/CT in head and neck cancer [44]. Compared to cervical MRI both PET tracers demonstrated slight diagnostic advantages, however, it should be taken into account that in almost half of patients (4/10), metal and/or movement artefacts severely impacted MRI image quality.

Presence of FAP on the cell surface of CAFs could be confirmed by IHC in all tumor specimens while it was absent in reactive, tumor-free lymph nodes. The high specificity of FAP-directed PET/CT may be particularly helpful in cases with extensive inflammatory changes in the oral cavity, in which [^18^F]FDG PET/CT faces challenges distinguishing malignant disease from non-specific changes. In this pilot study, only one false-positive lymph node was described in [^68^ Ga]FAPI-04 PET/CT, as compared to three in [^18^F]FDG PET/CT and five false-positives in MRI, respectively. Thus, by minimizing the number of neck dissections due to false-positive cervical lymph nodes, the extent of surgery might be individually tailored and morbidity significantly reduced without jeopardizing oncologic results. However, in view of relatively high physiological tracer uptake of [^68^ Ga]FAPI-04 in the oral mucosa [45], and increasing evidence for accumulation also in non-malignant inflammatory processes such as IgG4-related diseases [32, 46], the preliminary results of this pilot study should be taken with caution.

Interestingly, FAP expression in the cytoplasm and / or on the cell surface of the tumor cells could be observed in a subset of samples. This might be a non-specific reaction, e.g. to keratinization products; however, epithelial cancer cell expression of FAP has been reported for a variety of cancer entities including oral squamous cell cancer [22, 47]. In OSCC, FAP expression could be correlated with tumor size, lymph-node metastasis and shorter overall survival [22]. Whereas the current sample size of our study precludes any firm conclusions, CAF-directed PET/CT imaging might not only address CAF but also directly the tumor cell and serve as a prognostic non-invasive biomarker.

Several limitations of this pilot study need to be mentioned. First, only a small number of patients could be included, thus limiting statistical power of our results. In the cohort analyzed, no distant metastases or second malignancies were encountered, therefore the value of FAP-directed PET/CT in these scenarios could not be investigated. Another limitation could result from physiological tracer uptake of [^68^ Ga]FAPI-04 in the oral mucosa, which is comparable to that of [^18^F]FDG [45] and might potentially translate into compromised tumor-to-background contrast. However, in our small pilot study no difficulties in distinguishing primary tumor from surrounding tissue were encountered. Last, since no follow-up is available, no conclusion on the prognostic power of the new imaging tool can be drawn and future studies have to investigate whether the reported negative prognostic impact of high FAP expression in OSCC can be non-invasively detected by FAP-directed PET/CT [22].

In contrast, the homogeneous patient cohort with newly diagnosed, treatment-naïve subjects, the direct comparison between [^18^F]FDG, cervical MRI and FAP-directed PET/CT as well as the stringent pathological work-up (of primary tumors and 434 lymph nodes) including IHC are strengths of the current work.

## Conclusion

Given the improved (although not perfect) specificity of FAP-targeted imaging compared with [^18^F]FDG, [^68^ Ga]FAPI-04 (or its successors) may have the potential to prevent overtreatment and to reduce patient morbidity by minimizing the number of neck dissections due to false-positive cervical lymph nodes. However, given the small number of patients enrolled in this pilot study, no firm conclusion can be drawn at the moment, and further evaluation should be based on future large, prospective, multi-center studies.

## Supplementary Information

Below is the link to the electronic supplementary material.

Supplementary file1 (DOCX 18 KB)

## Data Availability

All data will be made available upon request.
