# Supplementary material for: Targeting fibroblast activation protein in newly diagnosed squamous cell carcinoma of the oral cavity – initial experience and comparison to [18F]FDG PET/CT and MRI
Source: Eur J Nucl Med Mol Imaging. 2021 May 29;48(12):3951–60. doi: 10.1007/s00259-021-05422-z (PMC8484183; doi:10.1007/s00259-021-05422-z)
Supplement: Supplementary file 1 — Supplementary file1 (DOCX 18 KB) [file 259_2021_5422_MOESM1_ESM.docx]

## Supplemental Table 1: Individual maximum (SUV_max_) and peak (SUV_peak_) maximum standardized uptake values for all primary tumors

|  |  | [^18^F]FDG PET | |  | FAP-directed PET | |
| --- | --- | --- | --- | --- | --- | --- |
| Pat # | **TU size [mm]** | **SUV_max_** | **SUV_peak_** |  | **SUV_max_** | **SUV_peak_** |
| 1 | 15 | 20.4 | 10.7 |  | 22.6 | 13.8 |
| 2 | 42 | 17.4 | 11.2 |  | 20.9 | 14.1 |
| 3 | 32 | 19.4 | 10.5 |  | 13.8 | 9.7 |
| 4 | 34 | 21.8 | 12.5 |  | 20.6 | 13.2 |
| 5 | 46 | 40.6 | 29.8 |  | 24.3 | 18.6 |
| 6 | 21 | 9.6 | 5.1 |  | 7.0 | 5.5 |
| 7 | 27 | 22.0 | 13.2 |  | 29.1 | 17.5 |
| 8 | 42 | 41.9 | 27.3 |  | 20.7 | 15.4 |
| 9 | 13 | 12.9 | 6.7 |  | 21.9 | 13.4 |
| 10 | 11 | 48.1 | 34.0 |  | 27.2 | 17.4 |

TU, Tumor; Pat, patient; SUV, standardized uptake value
